# Disseminated sporotrichosis caused by *Sporothrix brasiliensis* with testicular involvement

**DOI:** 10.1016/j.mmcr.2025.100716

**Published:** 2025-06-30

**Authors:** Regielly Caroline Raimundo Cognialli, Matheus Hideki Taborda, Bruno Hassunuma Carneiro, Carolina Melchior do Prado, Mariana Marques Wolski, Giovanni Luis Breda, Bram Spruijtenburg, Vânia Aparecida Vicente, Eelco F.J. Meijer, Flávio de Queiroz-Telles

**Affiliations:** a Hospital de Clínicas, Federal University of Paraná, Curitiba, Brazil; b Postgraduate Program in Microbiology, Parasitology and Pathology, Federal University of Paraná, Curitiba, Brazil; c Postgraduate Program in Internal Medicine and Health Sciences, Federal University of Paraná, Curitiba, Brazil; d Department of Medical Microbiology, Radboudumc, Nijmegen, the Netherlands; e Radboudumc-CWZ Center of Expertise for Mycology, Nijmegen, the Netherlands; f Department of Medical Microbiology and Immunology, Canisius-Wilhelmina Hospital (CWZ)/Dicoon, Nijmegen, the Netherlands; g Basic Pathology Department, Federal University of Paraná, Curitiba, Brazil; h Department of Public Health, Federal University of Paraná, Curitiba, Brazil

**Keywords:** *Sporothrix brasiliensis*, Immunocompromised, Disseminated, Testicular

## Abstract

Sporotrichosis is a growing public health concern especially due *Sporothrix brasiliensis*. We report a case of disseminated sporotrichosis involving the meninges, lungs, and testicles in a 35-year-old homeless man from Curitiba, Brazil. Fungal culture, histopathology, and molecular identification confirmed *S. brasiliensis* infection. Notably, testicular involvement—a rare complication of sporotrichosis—was initially misdiagnosed as a malignant tumor, leading to orchiectomy. A literature review identified only four previous cases of testicular sporotrichosis. This case underscores the potential for atypical presentations in immunocompromised patients and highlights the need for clinical vigilance in endemic regions, given the rising incidence of sporotrichosis.

## 1 Introduction

Sporotrichosis is a neglected disease and the most prevalent implantation mycosis worldwide [1]. In recent decades, *Sporothrix brasiliensis* has emerged as a significant pathogen, causing large outbreaks in Brazil and spreading to neighboring countries [1]. Transmission of *S. brasiliensis* occurs primarily through zoonotic routes, by infected cats through bites, scratches, exudates, or respiratory droplets [2]. However, it can also be transmitted through sapronotic routes by exposure to plants or debris and potentially through fomites [1]. Nevertheless, as the outbreak of *S. brasiliensis* continues to increase, atypical manifestations have increasingly been reported, including ocular, meningeal, osteoarticular, and pulmonary involvement [3].

We present a case of disseminated sporotrichosis caused by *S*. *brasiliensis* in an immunocompromised patient, with meningeal, pulmonary, and testicular involvement, along with a literature review.

## 2 Case

**Fig. 1:**
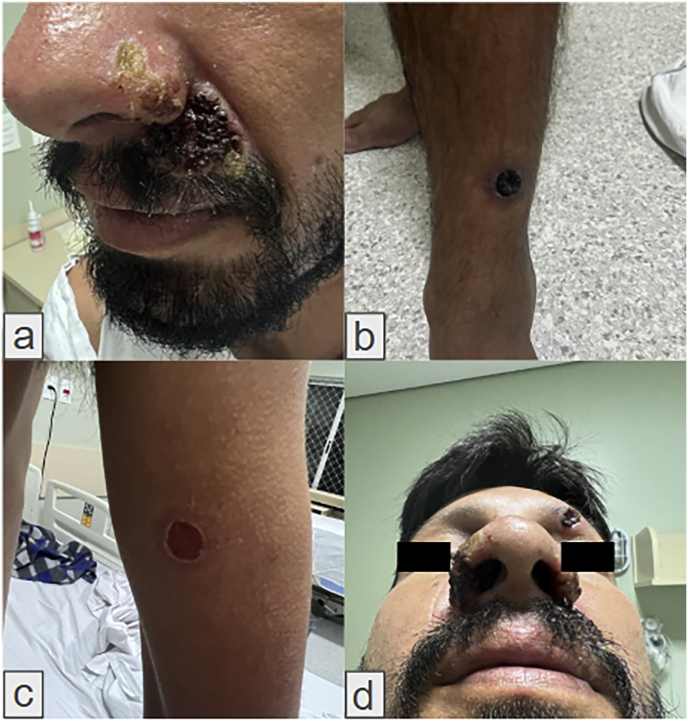
Clinical features. (a) meliceric crust on the ala nasi and crusts formed from coalescing pustules on the upper lip; (b) Ulcer with raised edges and necrotic center on the anterior surface of the left ankle; (c) Ulcer on the posterior aspect of the right arm with an erythematous center and raised edges, approximately 1,5 cm in diameter (d) Pustules on the ala nasi bilaterally.

A 35-year-old homeless man from Curitiba, Paraná, Brazil, presented to the emergency department (Day 0/D0) with a one-month history of disseminated ulcerated skin and mucosal lesions and persistent fever (Fig. 1). The patient had a history of drug abuse and was diagnosed with HIV in 2019. He reported irregular use of antiretroviral therapy and abandoned treatment in 2021, with a recent viral load of 84,369 copies/mL and a CD4 count of 257 cells/μL.

**Fig. 2:**
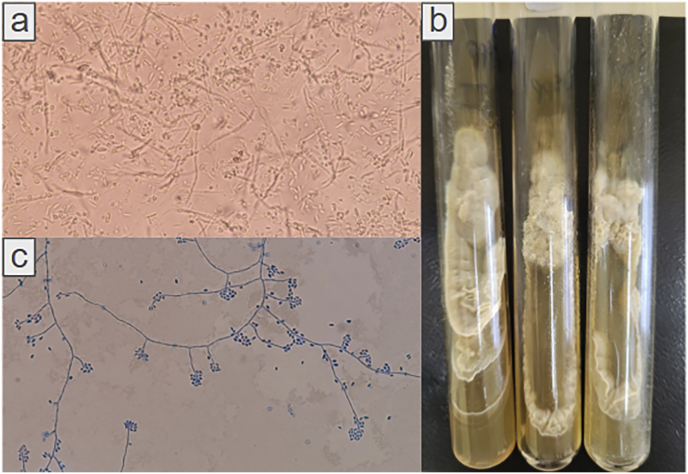
Microbiological findings. (a) Direct examination with potassium hydroxide (40 %) from arm biopsy showing a large quantitative of elongated yeast cells, some forming pseudohyphae (400x); (b) Culture of *Sporothrix* spp. from biopsies and blood culture showing beige membranous colonies on Sabouraud dextrose medium; (c) Micromorphology showing hyphae with sympodial conidiogenesis that resemble a flower (400x).

**Fig. 3:**
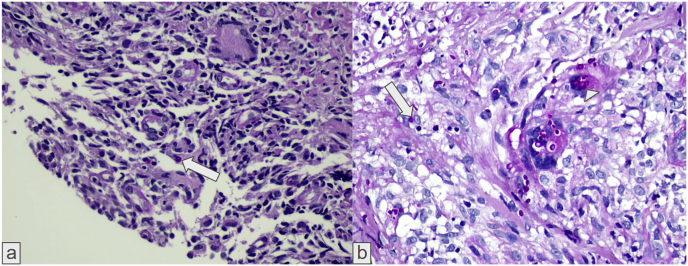
Histopathology. Mucosal (a) and skin (b) biopsies stained with periodic-acid Schiff (PAS) with digestion magnified 400x, demonstrating numerous *Sporothrix* sp. yeasts (arrows), some of which inside giant multinucleated cells (arrowhead), among chronic granulomatous inflammation.

Direct examination of skin biopsies from the left arm and palatal mucosa revealed a large number of elongated yeast cells, measuring 2–6 μm in diameter (Fig. 2A). Fungal cultures on Mycosel and Sabouraud dextrose agar at 30 °C showed growth of finely wrinkled, beige colonies after 3 days of incubation, which were morphologically identified as *Sporothrix* spp. (Fig. 2B and C). Histopathology examination also showed yeast cells (Fig. 3). Due to persistent fever, a blood culture was collected in a Myco/F Lytic medium, with growth detected after 7 days of incubation, also identified as *Sporothrix* spp. During hospitalization, the patient reported contact with stray animals and occasional work as a gardener. The fungal isolates were deposited at Microbiological Collections of the Paraná Network – Taxonline (CMRP/Taxonline- https://www.cmrp-taxonline.com/) under the accession numbers: CMRP5835, CMRP5788 and CMRP5834.

**Fig. 4:**
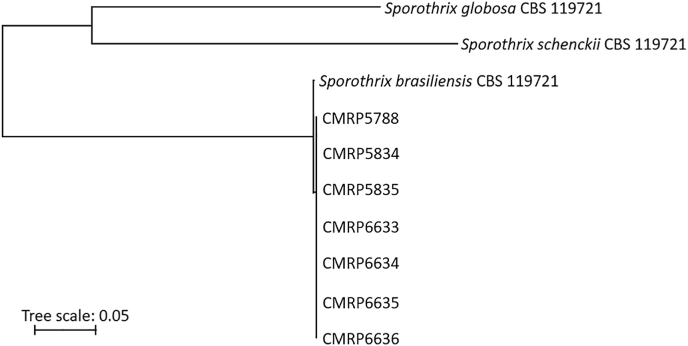
Phylogenetic tree of *S*. *brasiliensis* isolates from present case based on multiple sequence alignment of calmodulin sequences. As control isolates, *S. brasiliensis* CBS 133017 (KP101458.1), *S. schenckii* CBS 117440 (KP101386.1), *S. globosa* CBS 129721 (KP101478.1) were used.

Molecular identification of the isolates was performed using calmodulin (*CaM*) sequencing, as previously described [4]. All the isolates were identified as *S. brasiliensis* (Fig. 4). Generated sequences were deposited in GenBank under accession numbers PV441416-PV441422.

**Fig. 5:**
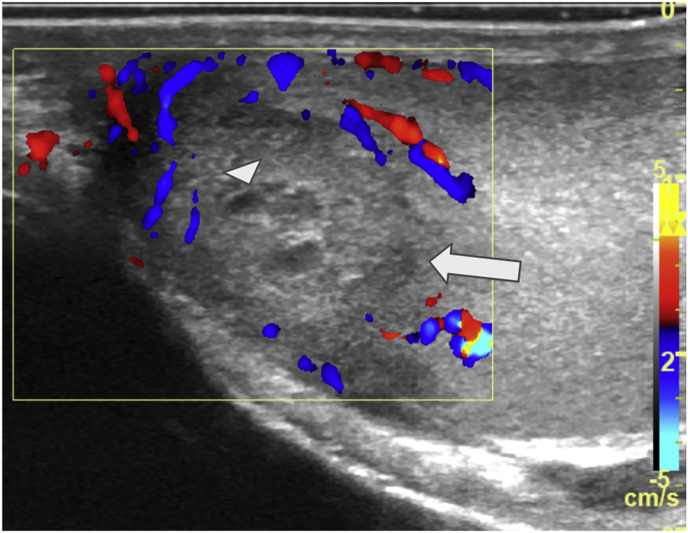
Testicular ultrasound - Isoechoic nodule with a hypoechoic halo (arrow) and a heterogeneous center (arrowhead). Doppler ultrasonography demonstrated peripheral vascularization.

During his hospital stay, the patient reported a previously unnoticed nodule on his right testicle. A scrotal ultrasound was performed, revealing findings suggestive of malignancy (Fig. 5). Consequently, an orchiectomy was conducted.

**Fig. 6:**
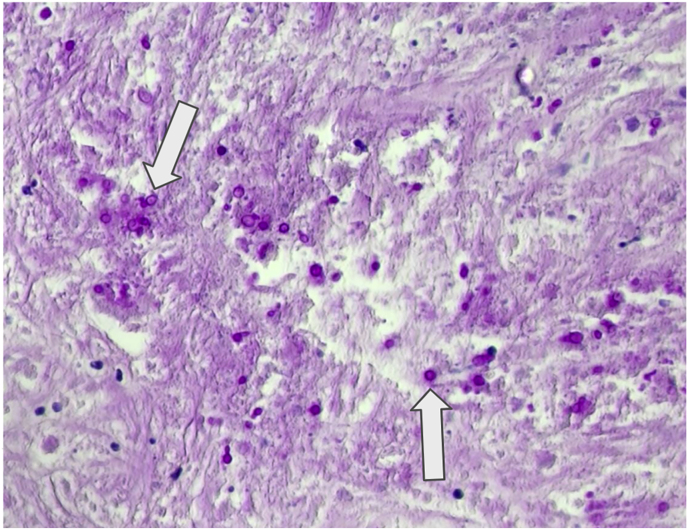
Testicular biopsy. Biopsy stained with PAS with diggestion and magnified 400x shows small round *Sporothrix* yeast (arrows) surrounded by chronic granulomatous inflammation with giant multinucleated cells and fibrosis.

However, histopathological examination of the testicle revealed necrotizing granulomatous inflammation and yeast cells consistent with *Sporothrix* infection (Fig. 6). Treatment with itraconazole (ITZ) (oral tablets 200 mg twice daily) was initiated. Although therapeutic drug monitoring for ITZ was unavailable at our institution, the patient nevertheless achieved resolution of fever and showed significant improvement of the lesions., The patient was discharged on D30 of hospitalization.

**Fig. 7:**
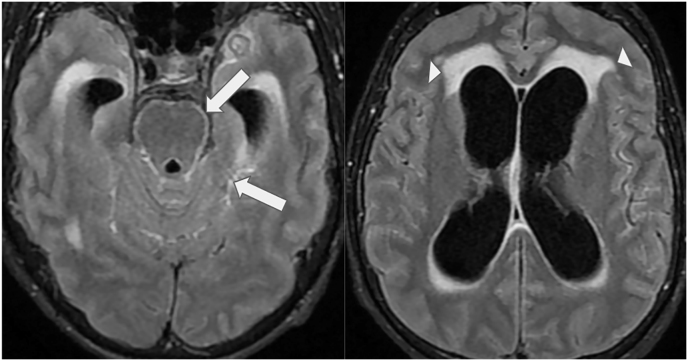
Brain MRI. Axial contrast-enhanced Fluid Attenuation Inversion Recovery (FLAIR) sequence shows linear areas of high signal intensity in the leptomeningeal surface of the brainstem and medial temporal cortex (arrows), enlarged supratentorial ventricles and adjacent transependymal white-matter edema (arrowheads).

The patient was readmitted on D92 with behavioral changes, recurrent fever, and anorexia. He reported regular use of antiretroviral therapy over the past two months. A computed tomography (CT) scan and magnetic resonance imaging (MRI) of the central nervous system (CNS) revealed hydrocephalus, leptomeningitis and transependymal edema (Fig. 7). Cerebrospinal fluid (CSF) analysis revealed an opening pressure of 25 cmH2O, with a cell count of 84.0 (97 % mononuclear cells), elevated protein levels (358.9 mg/dL), elevated lactate (6,67 mmol/L) and low glucose levels (22 mg/dL). Cryptococcal antigen testing by lateral flow assay was negative, PCR for viral meningitis and tuberculosis were also negative. CSF fungal and bacterial culture did not grow any microorganisms. Other opportunistic infections were also ruled out. A diagnosis of probable meningeal sporotrichosis was made. The patient underwent ventriculoperitoneal shunt placement, and a 30-day course of Amphotericin B (AMB) Lipid Complex (3 mg/kg) was initiated, resulting in significant clinical improvement. He was discharged on ITZ 200 mg/day (D124) and maintained outpatient follow-up.

The patient presented to the emergency department over one year after initial hospitalization (D397) with progressive worsening of right upper limb lesions and persistent fever. He reported initial clinical improvement after D124 discharge and maintained medication adherence for six months (until approximately D310), after which he discontinued all treatments. During this hospitalization, HIV viral load was 1,010,000 copies/mL, and his CD4 count was 363 cells/μL. Skin and nostril biopsies were collected, and fungal cultures revealed *Sporothrix* spp. growth. A chest CT scan showed a micronodular pattern suggestive of hematogenous dissemination. Given the clinical context, tuberculosis was considered in the differential diagnosis. Bronchoalveolar lavage and a pulmonary biopsy were performed. A rapid molecular test for tuberculosis yielded a negative result; however, fungal cultures from both specimens confirmed growth of *Sporothrix* spp. Those isolates were deposited at CMRP as: CMRP6633, CMRP6634, CMRP6635 and CMRP6636. The patient completed a 49-day course of AMB at a dose of 3 mg/kg, which resulted in clinical improvement. He was discharged on ITZ 400 mg daily (D449). One year later, he has had no disease relapses and has been able to maintain an undetectable HIV viral load.

**Fig. 8:**
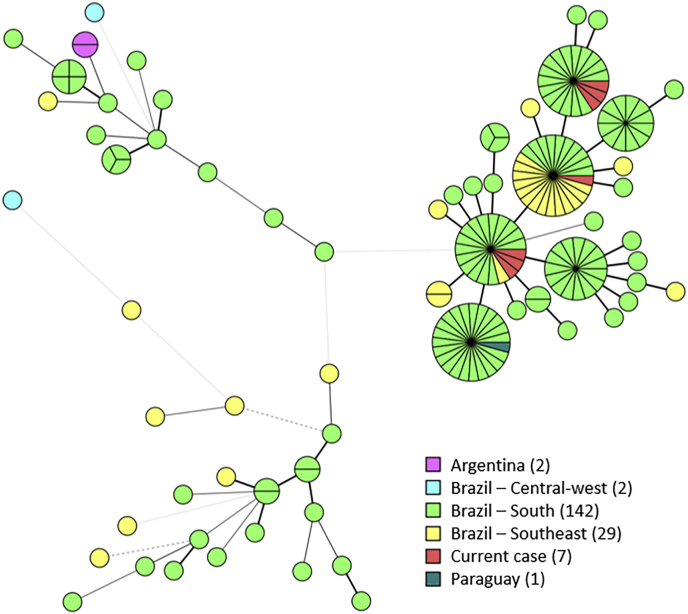
Minimum-spanning tree of 183 *S*. *brasiliensis* isolates, including seven from the current patient. Branch lengths indicate the similarity between genotypes with thick solid lines (variation in one marker), thin solid lines (variation in two markers), thin dashed lines (variation in three markers) and thin dotted lines (variation in four or more markers). Isolates colored according to origin with number of isolates in the legend.

Short tandem repeat (STR) genotyping was performed as described earlier and results were compared to previously genotyped isolates (Fig. 8) [5,6]. Three highly related genotypes were found that differed in one copy number for one marker between each other.

Antifungal susceptibility testing (AFST) according to the clinical laboratory standards institute (CLSI) M38 guidelines with modification as specified previously was conducted [6]. When minimum inhibitory concentrations (MICs) were compared to available tentative epidemiological cutoff values (ECVs), all isolates were classified as wild type [7] (Table 1).

**Table 1:** MICs of the *S*. *brasiliensis* isolates from the present case in the mycelial phase according to CLSI M38 guideline. Concentrations in μg/mL.

ID	AMB	FLU	ITZ	VOR	POS	ISA	AFG	MFG
CMRP5788	2	≥64	0.25	≥16	0.25	8	≤0.008	≤0.008
CMRP5834	2	≥64	0.25	≥16	0.5	≥16	≤0.008	≤0.008
CMRP5836	1	≥64	0.5	≥16	0.5	8	≤0.008	≤0.008
CMRP6633	2	≥64	0.5	≥16	0.25	≥16	≤0.008	≤0.008
CMRP6634	1	≥64	0.25	≥16	0.25	≥16	≤0.008	≤0.008
CMRP6635	1	≥64	0.5	≥16	0.25	8	≤0.008	≤0.008
CMRP6636	2	≥64	0.5	≥16	0.5	8	≤0.008	≤0.008

Legend: AMB - Amphotericin B; FLU - Fluconazole; ITZ - Itraconazole; VOR - Voriconazole; POS - Posaconazole; ISA - Isavuconazole; AFG - Anidulafungin; MFG - Micafungin. ∗For AFG and MFG the minimum effective concentration (MEC) was determined.

## 3 Discussion

Disseminated or extracutaneous sporotrichosis accounts for approximately 4 % of cases and is usually associated with immunocompromed conditions, including HIV/AIDS, alcoholism and diabetes mellitus [8]. In HIV infected patients, meningeal involvement is observed in nearly 17 % of cases, typically following skin lesions, and other organs may be affected [8,9]. Besides, there is a correlation between CD4 cell count and clinical manifestations, with more severe presentations occurring at lower CD4 levels [10]. A study evaluating a cohort of 80 patients with sporotrichosis observed that the mortality rate among HIV-positive patients was 62.5 % [9]. Therefore, atypical manifestations of sporotrichosis should be considered as opportunistic infections in HIV patients, particularly in endemic areas [10].

Testicular involvement in sporotrichosis remains rare, with only four cases reported in the literature (Table 2), none of which focused on its imaging characteristics. All cases were reported in the Americas: two in Brazil, one in Mexico, and one in the United States of America (USA). The mean age of the patients was 36 years (range: 27 to 54). All patients had disseminated sporotrichosis with concurrent skin and testicular involvement, and three also presented with meningeal disease. Other organs were affected in two cases. Only one patient had HIV/AIDS. One patient underwent orchiectomy due to suspicion of a malignant tumor, which was later ruled out and confirmed as sporotrichosis through histopathological examination. Molecular identification and antifungal susceptibility testing were not performed in any of the cases. Amphotericin B was the therapy of choice for all patients, administered as monotherapy in two cases and in combination with 5-flucytosine (5FC) or itraconazole in the other two cases. Three patients died due to complications such as multiple organ failure and septic shock.

**Table 2:** Summary of sporotrichosis cases with testicular involvement.

Reference	Year	Country	Age	Disseminated	Organs affected	HIV	Orchiectomy	Species ID	Molecular ID	AFST	Antifungal therapy	Outcome	Comments
[11]	1982	USA	27	Yes	Skin, testicle, meningeal	No	No	*Sporothrix* spp.	No	No	AMB+5FC	Cure	Drug user
[12]	2005	Brazil	29	Yes	Skin, testicle, meningeal, pancreas, bone marrow	Yes	No	*Sporothrix* spp.	No	No	ITZ + AMB	Death	–
[13]	2014	Mexico	54	Yes	Skin, testicle	No	Yes	*Sporothrix* spp.	No	No	AMB	Death	Alcoholism
[14]	2019	Brazil	36	Yes	Skin, testicle, meningeal/cerebral, kidney, liver, prostate, spleen	No	No	*Sporothrix* spp.	No	No	AMB	Death	Alcoholism, drug user, syphilis
Present case	2025	Brazil	35	Yes	Skin, testicle, meningeal, mucosal, lungs	Yes	Yes	*S. brasiliensis*	Yes	Yes	ITZ + AMB	Cure	Alcoholism, drug user

Similar to our case, one patient underwent a right radical orchiectomy for a suspected malignant tumor, but no imaging findings were described [13]. Ultrasound is the primary imaging modality for evaluating testicular masses and can identify focal lesions. However, a definitive diagnosis requires orchiectomy and histopathological analysis. In patients with disseminated sporotrichosis, as seen in all reported cases of testicular involvement, testicular sporotrichosis should be included in the differential diagnosis. In the other reported cases, the etiological agent was identified as *S. schenckii*; however, molecular identification was not performed in any of these cases, and the epidemiological sources of infection were not reported. This includes the two cases reported in Brazil, where no epidemiological link to zoonotic sporotrichosis was established In the present case, the patient reported exposure to stray animals and occupational history as a gardener. Since *S. brasiliensis* can be transmitted through both zoonotic and sapronotic routes, we cannot definitively establish the exact epidemiological link in this particular case. To our knowledge, this is the first reported case of disseminated sporotrichosis with testicular involvement caused by *S. brasiliensis.*

As many opportunistic infections must be considered in immunocompromised patients, laboratory diagnosis is essential. Gold standard for diagnosis of sporotrichosis is fungal culture, as direct examination and histopathological studies generally have low sensitivity for human cases [15]. However, as demonstrated in this case, the initial diagnosis was established through direct examination, due to the high fungal burden observed in the patient. Besides, initial growth of *Sporothrix* was detected after three days of incubation, enabling a rapid proven diagnosis. Species identification is performed using molecular techniques, such as partial sequencing of the *CaM* gene [15].

Among the species of the pathogenic clade, *S. brasiliensis* exhibits greater virulence, primarily attributed to its ability to form biofilms, melanin production, dimorphism, thermotolerance, metabolic plasticity and hemolytic activity [15]. This species is frequently associated with more severe cases and atypical clinical manifestations [3]. In the present case, whole genome sequencing is needed to confirm whether all isolates belong to the same strain and underwent microevolution in the STR markers within the host (presumed likely), or if there was a coinfection by different strains. Isolates belonged to a previously identified clonal clade that is present in the south and southeast of Brazil, indicating that the infection was probably acquired locally [5].

Isolation of *Sporothrix* spp. in blood cultures is rare and confirms hematogenous dissemination. A retrospective study involving 21 HIV-infected patients with sporotrichosis reported only one case with a positive blood culture from a patient with disseminated cutaneous disease [16]. Although blood culture has a low sensitivity for detecting *Sporothrix*, it is crucial to collect samples in enriched media such as Myco/F Lytic vials to improve diagnostic yield.

Diagnosis of meningeal sporotrichosis is challenging due to the similarity of signs and symptoms to those of other chronic causes of meningitis and the uncommon isolation of *Sporothrix* spp. in CSF [9,17]. A retrospective study conducted at a reference center reviewed all cases of disseminated sporotrichosis with neurological symptoms and identified 17 cases, with *Sporothrix* isolated in 41.2 % of CSF cultures [17]. Although the literature reports the isolation of *Sporothrix* in CSF, in the present case the patient had previously received antifungal treatment, which may have reduced the sensitivity of the culture. Given these challenges, it is essential to define a probable case definition of meningeal sporotrichosis when culture is negative. This could be based on the following criteria: patient with proven sporotrichosis presenting with meningeal symptoms, CSF analysis showing moderate pleocytosis (with mononuclear predominance), hyperproteinorrachia, hypoglycorrhachia, CSF negative for other microorganisms, and exclusion of other potential conditions. Panfungal PCR and 1,3-β-D-glucan testing remain underexplored for sporotrichosis diagnosis and were unavailable in our institution; however, these tools warrant further evaluation for clinical use in such cases.

Another rare manifestation is pulmonary sporotrichosis (PS), accounting for only 0.27 % of cases in hyperendemic areas such as Rio de Janeiro, Brazil [18]. PS can present as either primary or multifocal disease, with the latter being more commonly observed in patients with disseminated sporotrichosis and underlying immunosuppressive conditions [18]. Therefore, it is crucial to emphasize that patients with sporotrichosis who present with nonspecific respiratory symptoms or clinical features mimicking chronic pulmonary tuberculosis should undergo a thorough differential diagnosis, including the consideration of pulmonary sporotrichosis.

The management of sporotrichosis consists of the administration of antifungal agents, in addition to non-pharmacological measures, including cryotherapy and local heat therapy [19]. The best available evidence on the treatment of cutaneous or lymphocutaneous disease is with itraconazole-based regimens [19]. Terbinafine, potassium iodide solution and posaconazole are reasonable alternatives and may also be useful in refractory cases [19]. There are no clinical trials addressing severe and disseminated forms. Nonetheless, according to Brazilian and international guidelines, AMB therapies, preferably lipid formulations, are recommended [20]. The duration of therapy for local mild disease is usually 3–6 months [19]. It is reasonable to stop antifungals one month after clinical resolution of the lesions. In contrast, severe, disseminated forms require longer periods of treatment, initially with 2–6 weeks of intravenous AMB, followed by oral ITZ for 12 months or longer, depending on clinical improvement and resolution of immunosuppression [20]. Under ideal conditions, strategies to optimize the bioavailability and improve the pharmacokinetics of ITZ for prolonged use in severe disease include the use of an oral solution, which has better absorption characteristics compared to the capsule formulation, as well as therapeutic drug monitoring. All of the previously reported cases of testicular sporotrichosis were treated with AMB, and one also received ITZ. Interestingly, one patient was treated with adjunctive 5FC, although its use is anecdotal and lacks sufficient data on efficacy both in vitro and in vivo. Likewise, although echinocandins may exhibit some in vitro activity against certain *Sporothrix* spp. isolates, there is insufficient data to recommend this class of antifungals.

An additional challenge in diagnosing and managing HIV co-infected patients is the occurrence of immune reconstitution inflammatory syndrome (IRIS) [8]. It generally refers to clinical and/or radiological manifestations consistent with an inflammatory process, which may present as unmasking of previously undiagnosed lesions or as a paradoxical worsening of known disease following the initiation of antiretroviral therapy. As mentioned above, CSF cultures are positive for *Sporothrix* spp. in less than half of meningeal sporotrichosis cases. Furthermore, neurological symptoms have also been reported to occur as a result of IRIS. This clinical dilemma often necessitates an empirical decision regarding the appropriate strategy: to administer glucocorticoids under the assumption that IRIS is driving the clinical deterioration, or to intensify antifungal treatment in case of disease relapse or refractory infection, as was assumed in this patient's case.

To conclude, disseminated sporotrichosis with testicular involvement represents a rare but clinically significant manifestation of *S. brasiliensis* infection, particularly in immunocompromised individuals. This case highlights the importance of early recognition and laboratory confirmation of atypical presentations for accurate diagnosis and targeted therapy, as misdiagnosis can lead to unnecessary surgical interventions and poor clinical outcomes.

## CRediT authorship contribution statement

**Regielly Caroline Raimundo Cognialli:** Writing – review & editing, Writing – original draft, Visualization, Methodology, Investigation. **Matheus Hideki Taborda:** Writing – review & editing, Writing – original draft, Visualization, Investigation. **Bruno Hassunuma Carneiro:** Writing – review & editing, Writing – original draft, Visualization, Investigation. **Carolina Melchior do Prado:** Writing – review & editing, Formal analysis. **Mariana Marques Wolski:** Writing – review & editing, Resources. **Giovanni Luis Breda:** Writing – review & editing, Investigation. **Bram Spruijtenburg:** Writing – review & editing, Investigation, Visualization. **Vânia Aparecida Vicente:** Writing – review & editing, Validation. **Eelco F.J. Meijer:** Writing – review & editing, Writing – original draft, Investigation. **Flávio de Queiroz-Telles:** Writing – review & editing, Writing – original draft, Supervision.

## Ethical statement

Consent to participate.

Informed consent was obtained from the patient for publication.

## Conflict of interest

EFJM received research grants from Mundipharma and Scynexis, is in the scientific advisory board for Pfizer, and has received speaker fees from Gilead Sciences. All other authors declare no conflict of interest.
